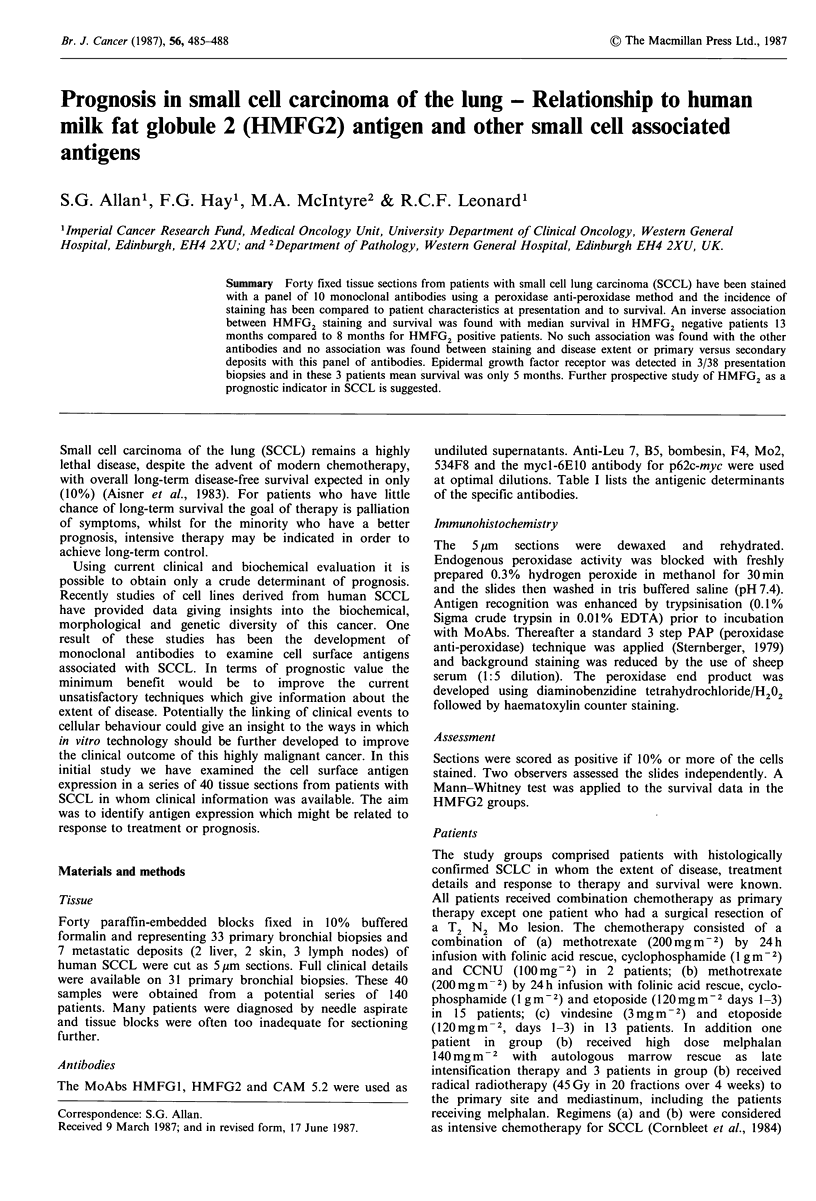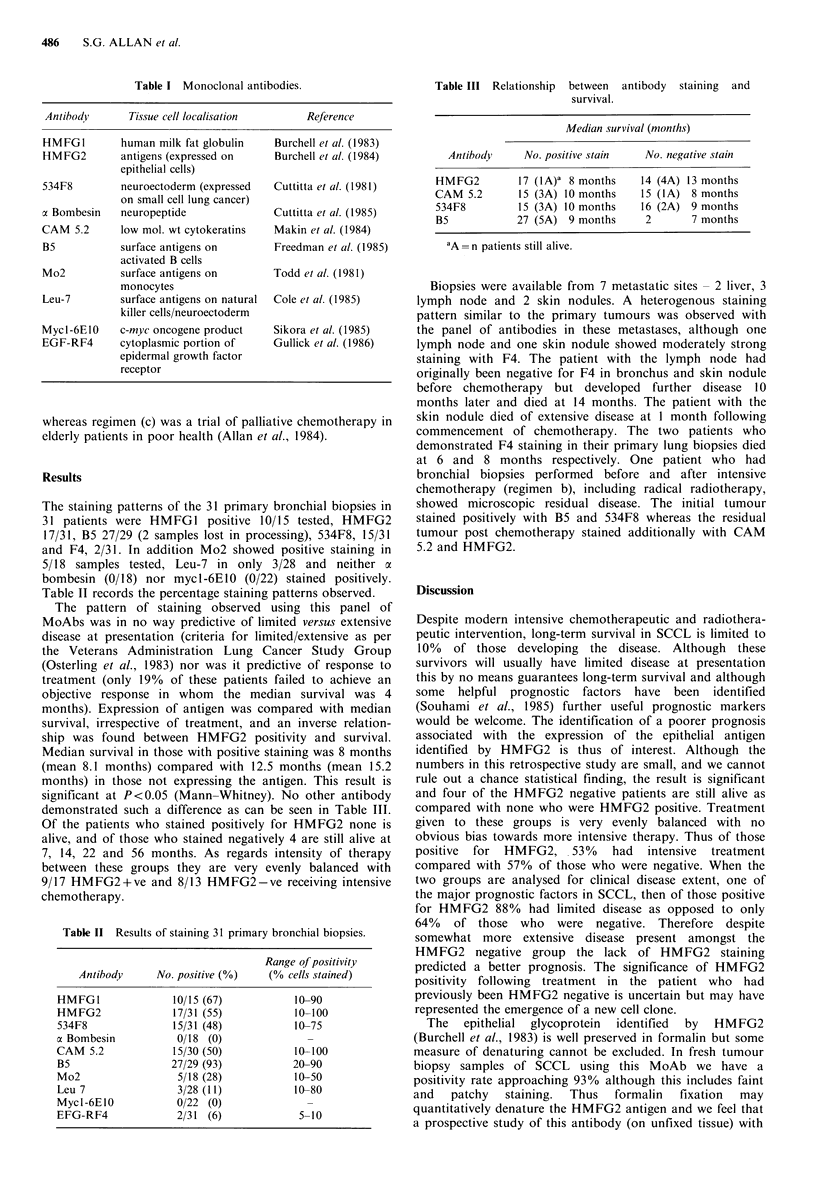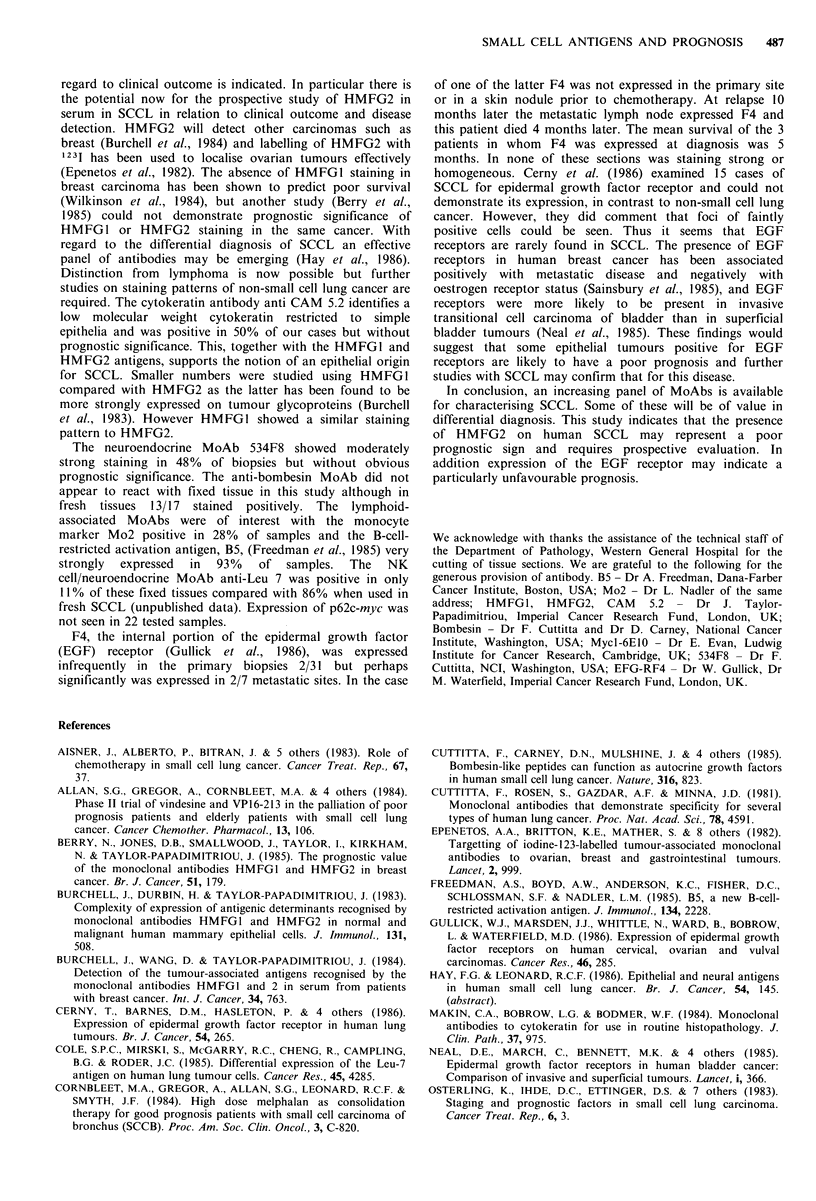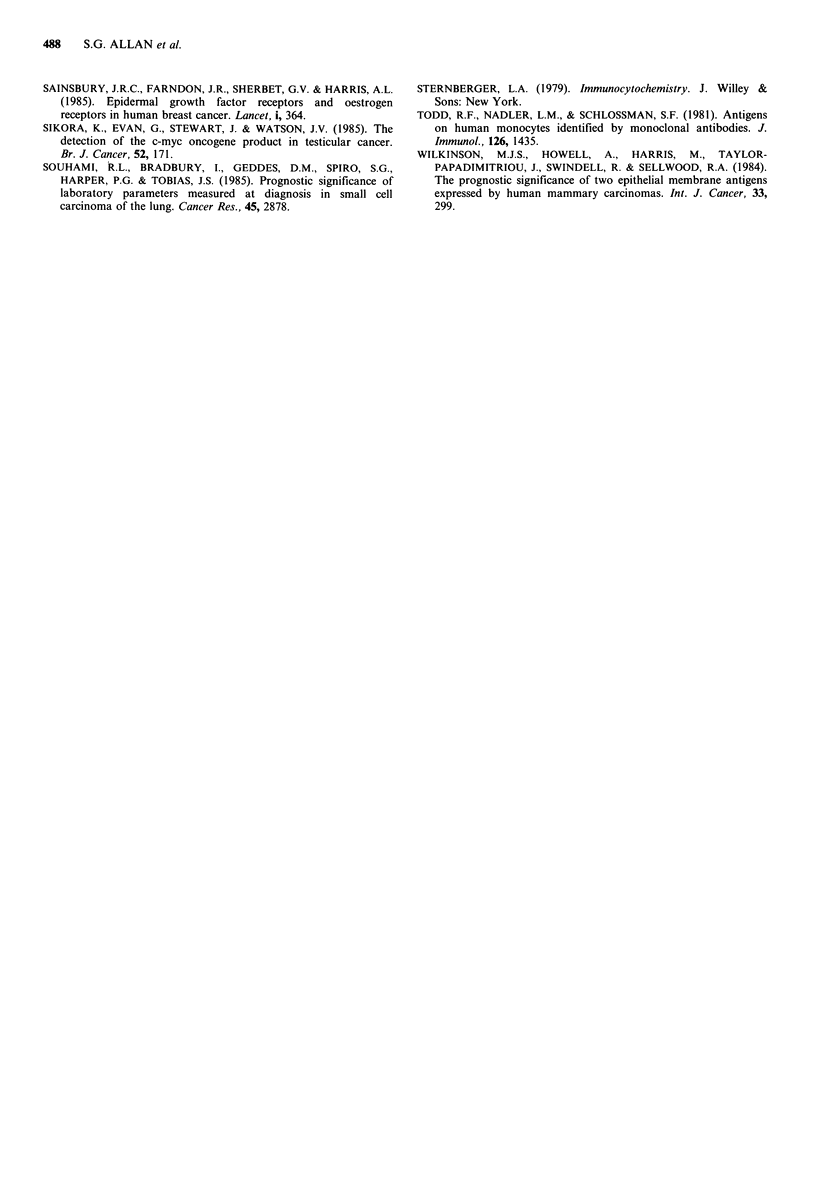# Prognosis in small cell carcinoma of the lung--relationship to human milk fat globule 2 (HMFG2) antigen and other small cell associated antigens.

**DOI:** 10.1038/bjc.1987.229

**Published:** 1987-10

**Authors:** S. G. Allan, F. G. Hay, M. A. McIntyre, R. C. Leonard

**Affiliations:** Imperial Cancer Research Fund, University Department of Clinical Oncology, Western General Hospital, Edinburgh, UK.

## Abstract

Forty fixed tissue sections from patients with small cell lung carcinoma (SCCL) have been stained with a panel of 10 monoclonal antibodies using a peroxidase anti-peroxidase method and the incidence of staining has been compared to patient characteristics at presentation and to survival. An inverse association between HMFG2 staining and survival was found with median survival in HMFG2 negative patients 13 months compared to 8 months for HMFG2 positive patients. No such association was found with the other antibodies and no association was found between staining and disease extent or primary versus secondary deposits with this panel of antibodies. Epidermal growth factor receptor was detected in 3/38 presentation biopsies and in these 3 patients mean survival was only 5 months. Further prospective study of HMFG2 as a prognostic indicator in SCCL is suggested.


Br. J. Cancer (1987), 56, 485–488 © The Macmillan Press Ltd., 1987

### Prognosis in small cell carcinoma of the lung – Relationship to human milk fat globule 2 (HMFG2) antigen and other small cell associated antigens

S.G. Allan[1], F.G. Hay[1], M.A. McIntyre[2] & R.C.F. Leonard[1]

[1]*Imperial Cancer Research Fund, Medical Oncology Unit, University Department of Clinical Oncology, Western General Hospital, Edinburgh, EH4 2XU; and* [2]*Department of Pathology, Western General Hospital, Edinburgh EH4 2XU, UK.*

**Summary** Forty fixed tissue sections from patients with small cell lung carcinoma (SCCL) have been stained with a panel of 10 monoclonal antibodies using a peroxidase anti-peroxidase method and the incidence of staining has been compared to patient characteristics at presentation and to survival. An inverse association between $HMFG_2$ staining and survival was found with median survival in $HMFG_2$ negative patients 13 months compared to 8 months for $HMFG_2$ positive patients. No such association was found with the other antibodies and no association was found between staining and disease extent or primary versus secondary deposits with this panel of antibodies. Epidermal growth factor receptor was detected in 3/38 presentation biopsies and in these 3 patients mean survival was only 5 months. Further prospective study of $HMFG_2$ as a prognostic indicator in SCCL is suggested.

Small cell carcinoma of the lung (SCCL) remains a highly lethal disease, despite the advent of modern chemotherapy, with overall long-term disease-free survival expected in only (10%) (Aisner *et al.*, 1983). For patients who have little chance of long-term survival the goal of therapy is palliation of symptoms, whilst for the minority who have a better prognosis, intensive therapy may be indicated in order to achieve long-term control.

Using current clinical and biochemical evaluation it is possible to obtain only a crude determinant of prognosis. Recently studies of cell lines derived from human SCCL have provided data giving insights into the biochemical, morphological and genetic diversity of this cancer. One result of these studies has been the development of monoclonal antibodies to examine cell surface antigens associated with SCCL. In terms of prognostic value the minimum benefit would be to improve the current unsatisfactory techniques which give information about the extent of disease. Potentially the linking of clinical events to cellular behaviour could give an insight to the ways in which *in vitro* technology should be further developed to improve the clinical outcome of this highly malignant cancer. In this initial study we have examined the cell surface antigen expression in a series of 40 tissue sections from patients with SCCL in whom clinical information was available. The aim was to identify antigen expression which might be related to response to treatment or prognosis.

### Materials and methods

### *Tissue*

Forty paraffin-embedded blocks fixed in 10% buffered formalin and representing 33 primary bronchial biopsies and 7 metastatic deposits (2 liver, 2 skin, 3 lymph nodes) of human SCCL were cut as 5 $\mu$m sections. Full clinical details were available on 31 primary bronchial biopsies. These 40 samples were obtained from a potential series of 140 patients. Many patients were diagnosed by needle aspirate and tissue blocks were often too inadequate for sectioning further.

### *Antibodies*

The MoAbs HMFG1, HMFG2 and CAM 5.2 were used as undiluted supernatants. Anti-Leu 7, B5, bombesin, F4, Mo2, 534F8 and the myc1-6E10 antibody for p62c-*myc* were used at optimal dilutions. Table I lists the antigenic determinants of the specific antibodies.

### *Immunohistochemistry*

The 5 $\mu$m sections were dewaxed and rehydrated. Endogenous peroxidase activity was blocked with freshly prepared 0.3% hydrogen peroxide in methanol for 30 min and the slides then washed in tris buffered saline (pH 7.4). Antigen recognition was enhanced by trypsinisation (0.1% Sigma crude trypsin in 0.01% EDTA) prior to incubation with MoAbs. Thereafter a standard 3 step PAP (peroxidase anti-peroxidase) technique was applied (Sternberger, 1979) and background staining was reduced by the use of sheep serum (1:5 dilution). The peroxidase end product was developed using diaminobenzidine tetrahydrochloride/$H_2 0_2$ followed by haematoxylin counter staining.

### *Assessment*

Sections were scored as positive if 10% or more of the cells stained. Two observers assessed the slides independently. A Mann–Whitney test was applied to the survival data in the HMFG2 groups.

### *Patients*

The study groups comprised patients with histologically confirmed SCLC in whom the extent of disease, treatment details and response to therapy and survival were known. All patients received combination chemotherapy as primary therapy except one patient who had a surgical resection of a $T_2$ $N_2$ Mo lesion. The chemotherapy consisted of a combination of (a) methotrexate (200 mg m$^{-2}$) by 24 h infusion with folinic acid rescue, cyclophosphamide (1 g m$^{-2}$) and CCNU (100 mg$^{-2}$) in 2 patients; (b) methotrexate (200 mg m$^{-2}$) by 24 h infusion with folinic acid rescue, cyclophosphamide (1 g m$^{-2}$) and etoposide (120 mg m$^{-2}$ days 1–3) in 15 patients; (c) vindesine (3 mg m$^{-2}$) and etoposide (120 mg m$^{-2}$, days 1–3) in 13 patients. In addition one patient in group (b) received high dose melphalan 140 mg m$^{-2}$ with autologous marrow rescue as late intensification therapy and 3 patients in group (b) received radical radiotherapy (45 Gy in 20 fractions over 4 weeks) to the primary site and mediastinum, including the patients receiving melphalan. Regimens (a) and (b) were considered as intensive chemotherapy for SCCL (Cornbleet *et al.*, 1984)

Correspondence: S.G. Allan.
Received 9 March 1987; and in revised form, 17 June 1987.

**Table I** Monoclonal antibodies.

| *Antibody* | *Tissue cell localisation* | *Reference* |
|---|---|---|
| HMFG1 | human milk fat globulin | Burchell *et al.* (1983) |
| HMFG2 | antigens (expressed on epithelial cells) | Burchell *et al.* (1984) |
| 534F8 | neuroectoderm (expressed on small cell lung cancer) | Cuttitta *et al.* (1981) |
| α Bombesin | neuropeptide | Cuttitta *et al.* (1985) |
| CAM 5.2 | low mol. wt cytokeratins | Makin *et al.* (1984) |
| B5 | surface antigens on activated B cells | Freedman *et al.* (1985) |
| Mo2 | surface antigens on monocytes | Todd *et al.* (1981) |
| Leu-7 | surface antigens on natural killer cells/neuroectoderm | Cole *et al.* (1985) |
| Mycl-6E10 | c-*myc* oncogene product | Sikora *et al.* (1985) |
| EGF-RF4 | cytoplasmic portion of epidermal growth factor receptor | Gullick *et al.* (1986) |

whereas regimen (c) was a trial of palliative chemotherapy in elderly patients in poor health (Allan *et al.*, 1984).

### Results

The staining patterns of the 31 primary bronchial biopsies in 31 patients were HMFG1 positive 10/15 tested, HMFG2 17/31, B5 27/29 (2 samples lost in processing), 534F8, 15/31 and F4, 2/31. In addition Mo2 showed positive staining in 5/18 samples tested, Leu-7 in only 3/28 and neither α bombesin (0/18) nor mycl-6E10 (0/22) stained positively. Table II records the percentage staining patterns observed.

The pattern of staining observed using this panel of MoAbs was in no way predictive of limited *versus* extensive disease at presentation (criteria for limited/extensive as per the Veterans Administration Lung Cancer Study Group (Osterling *et al.*, 1983) nor was it predictive of response to treatment (only 19% of these patients failed to achieve an objective response in whom the median survival was 4 months). Expression of antigen was compared with median survival, irrespective of treatment, and an inverse relationship was found between HMFG2 positivity and survival. Median survival in those with positive staining was 8 months (mean 8.1 months) compared with 12.5 months (mean 15.2 months) in those not expressing the antigen. This result is significant at $P<0.05$ (Mann–Whitney). No other antibody demonstrated such a difference as can be seen in Table III. Of the patients who stained positively for HMFG2 none is alive, and of those who stained negatively 4 are still alive at 7, 14, 22 and 56 months. As regards intensity of therapy between these groups they are very evenly balanced with 9/17 HMFG2+ve and 8/13 HMFG2−ve receiving intensive chemotherapy.

**Table II** Results of staining 31 primary bronchial biopsies.

| *Antibody* | *No. positive* (%) | *Range of positivity* (*% cells stained*) |
|---|---|---|
| HMFG1 | 10/15 (67) | 10–90 |
| HMFG2 | 17/31 (55) | 10–100 |
| 534F8 | 15/31 (48) | 10–75 |
| α Bombesin | 0/18 (0) | – |
| CAM 5.2 | 15/30 (50) | 10–100 |
| B5 | 27/29 (93) | 20–90 |
| Mo2 | 5/18 (28) | 10–50 |
| Leu 7 | 3/28 (11) | 10–80 |
| Mycl-6E10 | 0/22 (0) | – |
| EFG-RF4 | 2/31 (6) | 5–10 |

**Table III** Relationship between antibody staining and survival.

| *Antibody* | *Median survival* (*months*) | |
|---|---|---|
| | *No. positive stain* | *No. negative stain* |
| HMFG2 | 17 (1A)[a] 8 months | 14 (4A) 13 months |
| CAM 5.2 | 15 (3A) 10 months | 15 (1A) 8 months |
| 534F8 | 15 (3A) 10 months | 16 (2A) 9 months |
| B5 | 27 (5A) 9 months | 2 7 months |

[a]A = n patients still alive.

Biopsies were available from 7 metastatic sites – 2 liver, 3 lymph node and 2 skin nodules. A heterogenous staining pattern similar to the primary tumours was observed with the panel of antibodies in these metastases, although one lymph node and one skin nodule showed moderately strong staining with F4. The patient with the lymph node had originally been negative for F4 in bronchus and skin nodule before chemotherapy but developed further disease 10 months later and died at 14 months. The patient with the skin nodule died of extensive disease at 1 month following commencement of chemotherapy. The two patients who demonstrated F4 staining in their primary lung biopsies died at 6 and 8 months respectively. One patient who had bronchial biopsies performed before and after intensive chemotherapy (regimen b), including radical radiotherapy, showed microscopic residual disease. The initial tumour stained positively with B5 and 534F8 whereas the residual tumour post chemotherapy stained additionally with CAM 5.2 and HMFG2.

### Discussion

Despite modern intensive chemotherapeutic and radiotherapeutic intervention, long-term survival in SCCL is limited to 10% of those developing the disease. Although these survivors will usually have limited disease at presentation this by no means guarantees long-term survival and although some helpful prognostic factors have been identified (Souhami *et al.*, 1985) further useful prognostic markers would be welcome. The identification of a poorer prognosis associated with the expression of the epithelial antigen identified by HMFG2 is thus of interest. Although the numbers in this retrospective study are small, and we cannot rule out a chance statistical finding, the result is significant and four of the HMFG2 negative patients are still alive as compared with none who were HMFG2 positive. Treatment given to these groups is very evenly balanced with no obvious bias towards more intensive therapy. Thus of those positive for HMFG2, 53% had intensive treatment compared with 57% of those who were negative. When the two groups are analysed for clinical disease extent, one of the major prognostic factors in SCCL, then of those positive for HMFG2 88% had limited disease as opposed to only 64% of those who were negative. Therefore despite somewhat more extensive disease present amongst the HMFG2 negative group the lack of HMFG2 staining predicted a better prognosis. The significance of HMFG2 positivity following treatment in the patient who had previously been HMFG2 negative is uncertain but may have represented the emergence of a new cell clone.

The epithelial glycoprotein identified by HMFG2 (Burchell *et al.*, 1983) is well preserved in formalin but some measure of denaturing cannot be excluded. In fresh tumour biopsy samples of SCCL using this MoAb we have a positivity rate approaching 93% although this includes faint and patchy staining. Thus formalin fixation may quantitatively denature the HMFG2 antigen and we feel that a prospective study of this antibody (on unfixed tissue) with regard to clinical outcome is indicated. In particular there is the potential now for the prospective study of HMFG2 in serum in SCCL in relation to clinical outcome and disease detection. HMFG2 will detect other carcinomas such as breast (Burchell *et al.*, 1984) and labelling of HMFG2 with $^{123}I$ has been used to localise ovarian tumours effectively (Epenetos *et al.*, 1982). The absence of HMFG1 staining in breast carcinoma has been shown to predict poor survival (Wilkinson *et al.*, 1984), but another study (Berry *et al.*, 1985) could not demonstrate prognostic significance of HMFG1 or HMFG2 staining in the same cancer. With regard to the differential diagnosis of SCCL an effective panel of antibodies may be emerging (Hay *et al.*, 1986). Distinction from lymphoma is now possible but further studies on staining patterns of non-small cell lung cancer are required. The cytokeratin antibody anti CAM 5.2 identifies a low molecular weight cytokeratin restricted to simple epithelia and was positive in 50% of our cases but without prognostic significance. This, together with the HMFG1 and HMFG2 antigens, supports the notion of an epithelial origin for SCCL. Smaller numbers were studied using HMFG1 compared with HMFG2 as the latter has been found to be more strongly expressed on tumour glycoproteins (Burchell *et al.*, 1983). However HMFG1 showed a similar staining pattern to HMFG2.

The neuroendocrine MoAb 534F8 showed moderately strong staining in 48% of biopsies but without obvious prognostic significance. The anti-bombesin MoAb did not appear to react with fixed tissue in this study although in fresh tissues 13/17 stained positively. The lymphoid-associated MoAbs were of interest with the monocyte marker Mo2 positive in 28% of samples and the B-cell-restricted activation antigen, B5, (Freedman *et al.*, 1985) very strongly expressed in 93% of samples. The NK cell/neuroendocrine MoAb anti-Leu 7 was positive in only 11% of these fixed tissues compared with 86% when used in fresh SCCL (unpublished data). Expression of p62c-*myc* was not seen in 22 tested samples.

F4, the internal portion of the epidermal growth factor (EGF) receptor (Gullick *et al.*, 1986), was expressed infrequently in the primary biopsies 2/31 but perhaps significantly was expressed in 2/7 metastatic sites. In the case of one of the latter F4 was not expressed in the primary site or in a skin nodule prior to chemotherapy. At relapse 10 months later the metastatic lymph node expressed F4 and this patient died 4 months later. The mean survival of the 3 patients in whom F4 was expressed at diagnosis was 5 months. In none of these sections was staining strong or homogeneous. Cerny *et al.* (1986) examined 15 cases of SCCL for epidermal growth factor receptor and could not demonstrate its expression, in contrast to non-small cell lung cancer. However, they did comment that foci of faintly positive cells could be seen. Thus it seems that EGF receptors are rarely found in SCCL. The presence of EGF receptors in human breast cancer has been associated positively with metastatic disease and negatively with oestrogen receptor status (Sainsbury *et al.*, 1985), and EGF receptors were more likely to be present in invasive transitional cell carcinoma of bladder than in superficial bladder tumours (Neal *et al.*, 1985). These findings would suggest that some epithelial tumours positive for EGF receptors are likely to have a poor prognosis and further studies with SCCL may confirm that for this disease.

In conclusion, an increasing panel of MoAbs is available for characterising SCCL. Some of these will be of value in differential diagnosis. This study indicates that the presence of HMFG2 on human SCCL may represent a poor prognostic sign and requires prospective evaluation. In addition expression of the EGF receptor may indicate a particularly unfavourable prognosis.

We acknowledge with thanks the assistance of the technical staff of the Department of Pathology, Western General Hospital for the cutting of tissue sections. We are grateful to the following for the generous provision of antibody. B5 – Dr A. Freedman, Dana-Farber Cancer Institute, Boston, USA; Mo2 – Dr L. Nadler of the same address; HMFG1, HMFG2, CAM 5.2 – Dr J. Taylor-Papadimitriou, Imperial Cancer Research Fund, London, UK; Bombesin – Dr F. Cuttitta and Dr D. Carney, National Cancer Institute, Washington, USA; Mycl-6E10 – Dr E. Evan, Ludwig Institute for Cancer Research, Cambridge, UK; 534F8 – Dr F. Cuttitta, NCI, Washington, USA; EFG-RF4 – Dr W. Gullick, Dr M. Waterfield, Imperial Cancer Research Fund, London, UK.

### References

AISNER, J., ALBERTO, P., BITRAN, J. & 5 others (1983). Role of chemotherapy in small cell lung cancer. *Cancer Treat. Rep.*, **67**, 37.

ALLAN, S.G., GREGOR, A., CORNBLEET, M.A. & 4 others (1984). Phase II trial of vindesine and VP16-213 in the palliation of poor prognosis patients and elderly patients with small cell lung cancer. *Cancer Chemother. Pharmacol.*, **13**, 106.

BERRY, N., JONES, D.B., SMALLWOOD, J., TAYLOR, I., KIRKHAM, N. & TAYLOR-PAPADIMITRIOU, J. (1985). The prognostic value of the monoclonal antibodies HMFG1 and HMFG2 in breast cancer. *Br. J. Cancer*, **51**, 179.

BURCHELL, J., DURBIN, H. & TAYLOR-PAPADIMITRIOU, J. (1983). Complexity of expression of antigenic determinants recognised by monoclonal antibodies HMFG1 and HMFG2 in normal and malignant human mammary epithelial cells. *J. Immunol.*, **131**, 508.

BURCHELL, J., WANG, D. & TAYLOR-PAPADIMITRIOU, J. (1984). Detection of the tumour-associated antigens recognised by the monoclonal antibodies HMFG1 and 2 in serum from patients with breast cancer. *Int. J. Cancer*, **34**, 763.

CERNY, T., BARNES, D.M., HASLETON, P. & 4 others (1986). Expression of epidermal growth factor receptor in human lung tumours. *Br. J. Cancer*, **54**, 265.

COLE, S.P.C., MIRSKI, S., McGARRY, R.C., CHENG, R., CAMPLING, B.G. & RODER, J.C. (1985). Differential expression of the Leu-7 antigen on human lung tumour cells. *Cancer Res.*, **45**, 4285.

CORNBLEET, M.A., GREGOR, A., ALLAN, S.G., LEONARD, R.C.F. & SMYTH, J.F. (1984). High dose melphalan as consolidation therapy for good prognosis patients with small cell carcinoma of bronchus (SCCB). *Proc. Am. Soc. Clin. Oncol.*, **3**, C-820.

CUTTITTA, F., CARNEY, D.N., MULSHINE, J. & 4 others (1985). Bombesin-like peptides can function as autocrine growth factors in human small cell lung cancer. *Nature*, **316**, 823.

CUTTITTA, F., ROSEN, S., GAZDAR, A.F. & MINNA, J.D. (1981). Monoclonal antibodies that demonstrate specificity for several types of human lung cancer. *Proc. Nat. Acad. Sci.*, **78**, 4591.

EPENETOS, A.A., BRITTON, K.E., MATHER, S. & 8 others (1982). Targetting of iodine-123-labelled tumour-associated monoclonal antibodies to ovarian, breast and gastrointestinal tumours. *Lancet*, **2**, 999.

FREEDMAN, A.S., BOYD, A.W., ANDERSON, K.C., FISHER, D.C., SCHLOSSMAN, S.F. & NADLER, L.M. (1985). B5, a new B-cell-restricted activation antigen. *J. Immunol.*, **134**, 2228.

GULLICK, W.J., MARSDEN, J.J., WHITTLE, N., WARD, B., BOBROW, L. & WATERFIELD, M.D. (1986). Expression of epidermal growth factor receptors on human cervical, ovarian and vulval carcinomas. *Cancer Res.*, **46**, 285.

HAY, F.G. & LEONARD, R.C.F. (1986). Epithelial and neural antigens in human small cell lung cancer. *Br. J. Cancer*, **54**, 145. (*abstract*).

MAKIN, C.A., BOBROW, L.G. & BODMER, W.F. (1984). Monoclonal antibodies to cytokeratin for use in routine histopathology. *J. Clin. Path.*, **37**, 975.

NEAL, D.E., MARCH, C., BENNETT, M.K. & 4 others (1985). Epidermal growth factor receptors in human bladder cancer: Comparison of invasive and superficial tumours. *Lancet*, **i**, 366.

OSTERLING, K., IHDE, D.C., ETTINGER, D.S. & 7 others (1983). Staging and prognostic factors in small cell lung carcinoma. *Cancer Treat. Rep.*, **6**, 3.

SAINSBURY, J.R.C., FARNDON, J.R., SHERBET, G.V. & HARRIS, A.L. (1985). Epidermal growth factor receptors and oestrogen receptors in human breast cancer. *Lancet*, **i**, 364.

SIKORA, K., EVAN, G., STEWART, J. & WATSON, J.V. (1985). The detection of the c-myc oncogene product in testicular cancer. *Br. J. Cancer*, **52**, 171.

SOUHAMI, R.L., BRADBURY, I., GEDDES, D.M., SPIRO, S.G., HARPER, P.G. & TOBIAS, J.S. (1985). Prognostic significance of laboratory parameters measured at diagnosis in small cell carcinoma of the lung. *Cancer Res.*, **45**, 2878.

STERNBERGER, L.A. (1979). *Immunocytochemistry*. J. Willey & Sons: New York.

TODD, R.F., NADLER, L.M., & SCHLOSSMAN, S.F. (1981). Antigens on human monocytes identified by monoclonal antibodies. *J. Immunol.*, **126**, 1435.

WILKINSON, M.J.S., HOWELL, A., HARRIS, M., TAYLOR-PAPADIMITRIOU, J., SWINDELL, R. & SELLWOOD, R.A. (1984). The prognostic significance of two epithelial membrane antigens expressed by human mammary carcinomas. *Int. J. Cancer*, **33**, 299.